# Comparative litter decomposability traits of selected native and exotic woody species from an urban environment of north-western Siwalik region, India

**DOI:** 10.1038/s41598-020-64576-2

**Published:** 2020-05-12

**Authors:** Meenu Patil, Abhishek Kumar, Pardeep Kumar, Navneet Kaur Cheema, Rupinder Kaur, Ramchand Bhatti, A. N. Singh

**Affiliations:** Department of Botany, Panjab University, Chandigarh, 160014 India

**Keywords:** Ecosystem ecology, Invasive species, Urban ecology

## Abstract

Exotic plants can potentially modify ecosystem functions like cycling of nutrients by adjusting their decomposition rates. However, these effects are largely unknown for urban ecosystems, though they act as reservoirs of exotic plants. The present study evaluated the decomposition rates of five native and five exotic (three invasive and two non-invasive) species by conducting the litter bag experiment. Our study, however, did not find any significant differences in overall decomposition rates of native and exotic species but decomposition rates were strongly correlated with initial chemical quality of the litter. Further, litter carbon, lignin to nitrogen ratio and carbon to nitrogen ratio seemed to be good predictors for decomposition rates in this study. Interestingly, invasive exotic species had higher decomposition rate while non-invasive exotic species showed a slower rate as compared to the native species. In conclusion, our study indicates that invasive exotic plants try to maintain a higher chemical quality of litter than native and non-invasive exotic species which promotes their rapid decomposition. Thus, the better chemical quality of litter may facilitate the naturalisation and invasion of exotic plants irrespective of their origin.

## Introduction

The biological invasion has been considered as the second major cause next to habitat loss for recent extinctions and threatening of biodiversity^1^. Invasive plants are not only known to alter the species richness of native plants and animals^2–4^ but also have multidimensional impacts on ecosystem structure and functions that can interfere with the stability of global ecosystems^5,6^. The invasion will likely increase with the current rate of climate change and globalisation^7,8^, though the climatic benefits may be highly species-specific^9^. Moreover, invasion can be a more serious problem for developing countries like India, where increasing trade will further risk the naturalisation of exotic plants^10^.

Among global ecosystems, urban ecosystems offer several invasion-facilitating factors such as human interference, favourable ecosystem properties and altered biotic interactions^11^, which make them prone to invasion. These ecosystems usually have peculiar environmental conditions such as climate (higher temperature), biodiversity (high proportion of exotics), a high degree of anthropogenic disturbance, distinct soils and bio-geo-chemistry^12^. Theoretically, the natural and anthropogenic mechanism may interact to drive invasion in the urban environment, and therefore, the unique set of biological and environmental conditions of these ecosystems might facilitate the invasion of exotic species^13^. Several hypotheses have been proposed to explain the invasion success of exotic plants^13–19^ and major factors that support invasion in the urban ecosystems are summarised as follows:

1. Exotic plants usually intentionally introduced several times into the urban environment for ornamental and horticultural purposes^12,20^. Thus, a higher propagule of exotic species can favour their naturalisation and invasion process. This is popularly called as “propagule pressure hypothesis” and widely supported in the literature available to date^14^.
2. Urban areas are probably having excellent opportunities for rapid dispersal of exotic species through a high human population and vehicular movements that can act as efficient dispersers in urban environments^15^.
3. These ecosystems have higher levels of disturbance that support the invasion of non-native species. There is growing evidence showing that urbanisation can be thought of as a proxy for disturbance, which may favour naturalisation and invasion of exotic plants^13,16^. This highly supported^14^ notion is called as the “disturbance hypothesis”^17^.
4. The unique environmental conditions of the urban ecosystems such as temperature, resource availability, habitat availability, etc. might be favouring the spread of invasive species. For example, the relatively higher temperature in urban ecosystems^12^ may promote the spread of tropical plants. Similarly, plants seem to benefit from higher resource availability especially in the form of managed gardens where a large number of exotic species usually flourish. Thus, higher nutrients can promote their naturalisation and invasion success, although resource availability in urban ecosystems can highly context- and species-specific.
5. Altered environmental conditions of the urban ecosystems may offer novel biotic interactions and it is expectable that reduced negative interactions (such as competition, predation) and increased positive interactions (such as facilitation, pollination) are going to benefit invasion of exotic species. For example, reduced competition for resources such as light and nutrients in the urban gardens and managed areas will benefit exotic plants due to abundant resources and lower species richness. This is why the invasion of weeds with high specific leaf area and profuse growth traits often erupt in these ecosystems. Similarly, whenever a species escapes its natural range, it also escapes from its natural predators and pathogens, which facilitates its invasion due to uncontrolled growth of the species. This mechanism of invasion is widely known as “enemy release (ER) hypothesis”^18^, though it is not always true for every invading species or ecosystem^11^. Generally, exotic pollinators may prefer exotic plants over native and therefore facilitate their invasion. Such facilitation of invasion by positive interactions among exotics is broadly supported^11^ and usually referred to as “invasion meltdown hypothesis”^19^.
6. Urban ecosystems have relatively lower biodiversity as compared to natural ecosystems and therefore are more susceptible to invasion. This is referred to as the “biotic resistance hypothesis”^17^, however, little evidence is available to support this hypothesis^21^.

Apart from numerous invasion-facilitation advantages provided by the urban environment, exotic plants may modify the ecosystem processes such as nutrient cycling, which might promote their fitness and growth. Thus, exotic plants may influence critical ecological processes like litter decomposition^22^, which primarily govern the cycling of nutrients^23^. Previous studies have shown that the litter of some exotic species may decompose more rapidly as compared to native species^23–25^, but opposite patterns have also been observed^6^. Furthermore, some studies report that exotic and native species do not differ in their litter decomposition rate at all^26,27^. Thus, it can be suggested that the decomposition rate of litter seems to be highly species-specific and it is still unclear why litter of some exotic plants decomposes at a higher or slower rate as compared to native plant litter. Further, the decomposition rate can be entirely different in urban ecosystems because the unique environmental conditions of these ecosystems may offer several advantages to the exotic species.

The differences in the rates of litter decomposition are highly influenced by climate (mainly temperature and precipitation), litter quality (in terms of physico-chemical traits) and the composition of decomposer organisms^28–30^. Though the combined effects of species traits and climatic conditions can explain a large proportion of variations in litter decomposition rates, factors responsible for the remaining proportion of variations are still unclear^29,30^. The effects of soil decomposer community are often considered to explain this residual variation in decomposition rates, however, their effects can be larger depending upon the context^31^. It has been speculated that the decomposer group may become specialised to decompose a particular type of litter of a species^32^ and therefore, expected to increase the decomposition rate of the litter receiving from the plants just above them. A considerable amount of evidence acknowledges that the litter of a species decomposes rapidly on a site where it has to be naturally fallen and decomposed as compared to other sites^32–34^. This effect is usually referred to as the “home-field advantage (HFA)”^35^, though it is not necessarily observed in all cases^36–39^. Thus, the abundance and activity of the decomposer groups may exert control over decomposition rates of the litter. Therefore, an easily degradable and digestible litter with high nutrient content will lead to the greater activity of the decomposer group, which will result in a faster decomposition rate. The efficiency of decomposers to degrade and digest the litter depending on the physico-chemical traits of the litter^40^. Therefore, several physico-chemical traits have been implicated for explaining the dominant controls and predictors of litter decomposition rates across different ecosystems^30^. For example, leaf dry matter content^41,42^, leaf pH^41^, leaf tensile strength (toughness)^43,44^, specific leaf area^45^, carbon (C)^46,47^, C/N ratio^27,48,49^, lignin (L)^50,51^, L/N ratio^52,53^ and nitrogen (N)^49,54^ have been suggested as dominant controller and predictors of the litter decomposition rates from various ecosystems. These studies indicate that a litter with characteristics like high tensile strength, high specific leaf area, low dry matter content, high nutrients (carbon, nitrogen, phosphorus) and low lignin tends to decompose faster because it will be easily degradable to decomposer group and therefore said to be of ‘high quality’^40,55^. Thus, the physico-chemical traits of the leaf litter might affect the rate of decomposition by influencing the activity of the soil decomposer group.

The city Chandigarh has a relatively smaller population size and a high GDP per capita^56^, making it highly susceptible to the spread of invasion^57^. It was established around 1960 and a large number of exotic plants were also introduced for ornamental and avenue purposes, despite the efforts to retain old indigenous trees. However, some of the exotic species have entered and dominated the nearby or adjacent forest patches forest areas of this urban ecosystem only during the past decades. Therefore, these forest patches are at high risk of invasion due to their suitability for entry and spread of exotic plants that may later turn into invasive species. Despite high invasion risks of urban forest patches, most of the previous studies have focused the natural terrestrial and aquatic systems for studies that aimed to investigate different aspects of biological invasion. Further, some of earlier decomposition studies have considered only a few native and exotic species for comparison that may potentially increase biasness^25,58^. To understand the possible mechanism of increasing abundance and to minimise impacts of exotic species, it becomes imperative to study the ecological traits (such as decomposability traits) of exotic species. We propose that exotic and native species may differ in their nutrient use efficiency, which should increase their fitness through changes in physico-chemical traits. The superior physico-chemical traits may help plants to produce high-quality litter which tend to enhance their decomposition rates and cycling of nutrients. These altered ecosystem processes might be favouring their increased abundance through a positive feedback mechanism. Therefore, the present study was designed to ask the following questions: (1) Are native and exotic species differ in their initial leaf litter chemistry? (2) Does the rate of decomposition is affected by the origin of species? (3) How invasive and non-invasive exotic plants differ in their litter chemistry or decomposability traits? (4) Can litter chemistry predict the rate of litter decomposition?

## Results

### Weight loss of leaf litter

**Figure 1:**
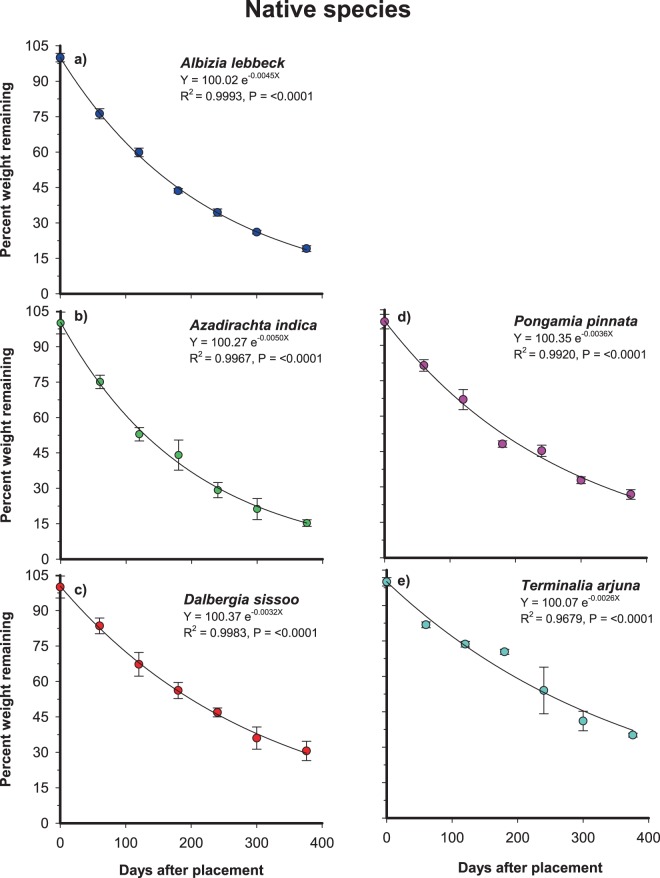
The exponential relationship between per cent weight loss of native woody species (**a**–**e**) leaf litter remaining and time elapsed since the placement of litterbags on the soil surface of the experimental site. Bars are 1 SD and the fitted line represents the regression equation.

**Figure 2:**
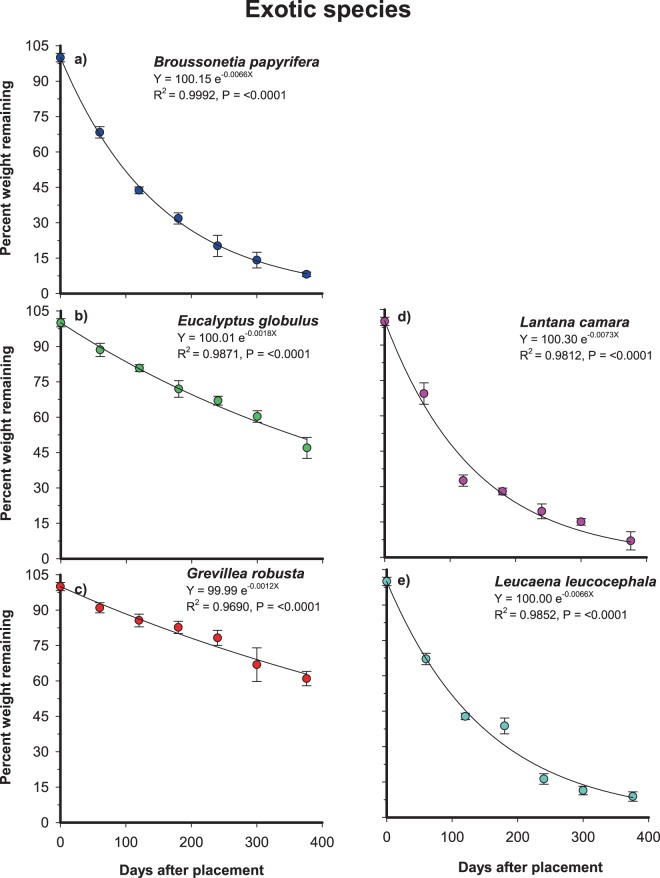
The exponential relationship between per cent weight loss of exotic woody species (**a**–**e**) leaf litter remaining and time elapsed since the placement of litterbags on the soil surface of the experimental site. Bars are 1 SD and the fitted line represents the regression equation.

The various litter decomposition parameters as evaluated by the litter bag technique are shown in Table 1. The pattern of weight loss among native species was similar in all the species and followed a single exponential relationship (Figs. 1 and 2). The slope value ranged from 0.0026–0.0050 with lowest value belonging to *T*. *arjuna* and highest to *A*. *indica* indicating that decomposition of leaf litter was most rapid in *A*. *indica* followed by *A*. *lebbeck*, *P*. *pinnata*, *D*. *sissoo* and *T*. *arjuna* (Fig. 1). The corresponding parameter for the exotic species was ranged from 0.0012–0.0073, being highest in *L*. *camara* and the lowest was found in *G*. *robusta* (Fig. 2). The same was also evident from Table 1 showing that annual decay constant was highest for *A*. *indica* (1.83 yr^−1^) and lowest for *T*. *arjuna* (1.02 yr^−1^) among native species whereas it was highest for *L*. *camara* (2.67 yr^−1^) and lowest for *G*. *robusta* (0.48 yr^−1^). Similarly, mean relative decomposition rate varied from 2.93 to 3.65 mg g^−1^ day^−1^, being highest for *A*. *indica* and lowest for *T*. *arjuna* among native species whereas the corresponding parameter among exotic species ranged from 1.62 to 3.88 mg g^−1^ day^−1^ being highest for *L*. *camara* and lowest for *G*. *robusta*. Two other decomposition parameters i.e. time taken for 50% and 95% weight loss also depicted the same pattern (Table 1). ANOVA indicated significant differences in weight loss due to species, origin and their interactions species × origin (Table 2).

**Table 1:** Leaf litter decomposition parameters of selected woody plant species. Values are means of three replicates (n = 3) with ± 1 SD. Mean values of native and exotic species in each column suffixed with the same letter are not significantly different from each other at *p* < 0.05 probability level.

Plant species	Decay constant (yr^−1^)	Mean relative decomposition rate (mg g^−1^ day^−1^)	Time for 50% decomposition (days)	Time for 95% decomposition (days)
**Native species**				
*Albizia lebbeck*	1.61 ± 0.05^a^	3.46 ± 0.02^a^	158.00 ± 5.00^a^	682.00 ± 22.00^a^
*Azadirachta indica*	1.83 ± 0.11^a^	3.65 ± 0.14^a^	139.00 ± 9.00^a^	601.00 ± 37.00^a^
*Dalbergia sissoo*	1.16 ± 0.11^a^	3.11 ± 0.13^a^	220.00 ± 21.00^b^	954.00 ± 92.00^b^
*Pongamia pinnata*	1.28 ± 0.09^a^	3.23 ± 0.15^a^	198.00 ± 14.00^a^	859.00 ± 62.00^b^
*Terminalia arjuna*	1.02 ± 0.02^a^	2.93 ± 0.04^ab^	249.00 ± 4.00^b^	1078.00 ± 16.00^ab^
**Exotic species**				
*Broussonetia papyrifera*	2.44 ± 0.10^b^	3.79 ± 0.01^c^	104.00 ± 4.00^b^	450.00 ± 19.00^a^
*Eucalyptus globulus*	0.74 ± 0.10^a^	2.44 ± 0.27^bc^	348.00 ± 48.00^c^	1506.00 ± 209.00^b^
*Grevillea robusta*	0.48 ± 0.06^a^	1.62 ± 0.27^a^	531.00 ± 63.00^d^	2299.00 ± 271.00^c^
*Lantana camara*	2.67 ± 0.48^b^	3.88 ± 0.08^c^	97.00 ± 20.00^a^	420.00 ± 84.00^a^
*Leucaena leucocephala*	2.37 ± 0.21^b^	3.79 ± 0.03^c^	107.00 ± 9.00^ab^	464.00 ± 41.00^a^

**Table 2:** Summary of ANOVA for the effect of species, origin and their interaction (Species × Origin) for leaf litter decomposition and decomposability traits of exotic and native woody species. A general linear model (~ Intercept + Species + Origin + Species: Origin) was used followed by Tukey’s HSD post-hoc test.

Parameters	Species		Origin		Species × Origin	
	*F*_4,20_	*p*	*F*_4,20_	*p*	*F*_4,20_	*p*
Nitrogen	75.535	0.000	88.367	0.000	54.586	0.000
Phosphorus	44.557	0.000	16.598	0.001	31.971	0.000
Lignin	13.597	0.000	17.485	0.000	11.775	0.000
Carbon	2.657	0.063	0.116	0.737	3.950	0.016
Cellulose	22.601	0.000	667.279	0.000	50.367	0.000
Crude fibre	12.128	0.000	0.009	0.927	8.462	0.000
L/C	11.049	0.000	18.354	0.000	8.605	0.000
L/N	42.218	0.000	16.144	0.001	31.425	0.000
L/P	46.417	0.000	8.551	0.008	48.790	0.000
C/N	132.309	0.000	45.445	0.000	100.495	0.000
C/P	73.744	0.000	6.599	0.018	87.261	0.000
N/P	4.571	0.009	7.015	0.015	6.372	0.002
*k*	70.041	0.000	28.308	0.000	34.666	0.000
MRD	108.901	0.000	9.989	0.005	52.144	0.000
T_50_	105.338	0.000	19.733	0.000	55.448	0.000
T_95_	105.303	0.000	19.823	0.000	55.330	0.000

*k* =  Decay constant, MRD = Mean relative decomposition, T50 = Time for 50% decomposition, T95 = Time for 95% decomposition.

### Litter decomposition parameters among exotic and native species

**Figure 3:**
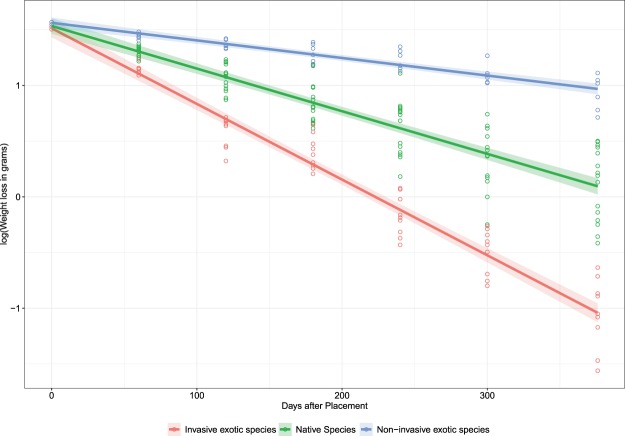
Leaf litter of invasive species (*Broussonetia papyrifera*, *Lantana camara* and *Leucaena leucocephala*) rapidly decomposed while exotic species (*Eucalyptus globulus* and *Grevillea robusta*) slowly decomposed their leaf litter as compared to native species.

In contrast to our expectations, the litter decomposition parameters (*k*, MRD, T_50_ and T_95_) did not significantly differ (*p* < 0.05) among selected native and exotic woody species (see Supplementary Table S1). This is also evident from an almost similar pattern of weight loss among native and exotic species (see Supplementary Fig. S1). But, the decay constant (*k*) was significantly lower (*p* = 0.03) for non-invasive exotic species and significantly higher (*p* < 0.05) for invasive exotic species as compared to native species (see Supplementary Table S1). The same is also evident from the decomposition rate pattern which is distinguishable among native, non-invasive exotic and invasive exotic species (Fig. 3, see Supplementary Fig. S2). Similarly, the mean relative decomposition rate of native and exotic species did not differ significantly, however, the MRD of non-invasive exotic species was significantly lower and that of invasive exotic species was significantly higher from native species. Moreover, the MRD of non-invasive exotic species was significantly lower than invasive exotic species (see Supplementary Table S1).

### Initial chemical characterization of leaf litter

The overall results of the initial chemical analyses of leaf litter are represented in Table 3. Among native species, nitrogen and cellulose content was highest for *P*. *pinnata* and lowest for *A*. *indica*. However, carbon to nitrogen and lignin to nitrogen ratios exhibited the opposite trend. The phosphorus content was also highest for *P*. *pinnata* but lowest in case of *D*. *sissoo*. The lowest phosphorus content in *D*. *sissoo* has resulted in the highest lignin to phosphorus and carbon to phosphorus ratio. The crude fibre content was lowest for *P*. *pinnata* but highest for *D*. *sissoo*. Further, lignin content and lignin to carbon ratio was highest for *A*. *lebbeck* and lowest for *P*. *pinnata*. The *D*. *sissoo* had the highest carbon content and nitrogen to phosphorus ratio but carbon content was lowest for *A*. *lebbeck* and nitrogen to phosphorus ratio was lowest for *T*. *arjuna* (Table 3). On the other hand, among exotic species, the nitrogen and phosphorus content was highest for *B*. *papyrifera* but lowest for *G*. *robusta* (Table 3). Further, *G*. *robusta* had the highest lignin and carbon content while *B*. *papyrifera* had the lowest lignin content. Consequently, lignin to carbon, lignin to nitrogen, lignin to phosphorus, carbon to nitrogen and carbon to phosphorus ratio was highest for *G*. *robusta*. However, these ratios showed the opposite trend for *B*. *papyrifera*. The *L*. *camara* had lowest cellulose content and highest crude fibre content while cellulose content was highest in *E*. *globulus* and crude fibre content was lowest in *G*. *robusta*. The carbon content was lowest in *B*. *papyrifera* and highest in *G*. *robusta*. The nitrogen to phosphorus ratio was lowest in *E*. *globulus* whereas highest for *B*. *papyrifera* (Table 3).

**Table 3:** Initial chemical characterization of selected woody species used for litter decomposition study. Values are means of three replicates (n = 3) ± 1 SD. The values in each column suffixed with the same letter are not significantly different from each other at *p* < 0.05 probability level when tested with a general linear model (~Species) followed by Tukey’s HSD post-hoc test.

Plant Species	Nitrogen (%)	Phosphorus (%)	Lignin (%)	Carbon (%)	Cellulose (%)	Crude fibre	L/C Ratio	L/N ratio	L/P ratio	C/N ratio	C/P ratio	N/P ratio
**Native species**												
*Albizia lebbeck*	1.47 ± 0.19^a^	0.10 ± 0.01^b^	24.49 ± 0.83^b^	42.50 ± 3.65^a^	26.30 ± 1.47^a^	25.33 ± 5.13^a^	0.57 ± 0.04^c^	16.87 ± 2.84^b^	241.78 ± 2.54^c^	29.21 ± 4.58^b^	419.83 ± 38.29^c^	14.60 ± 2.44^b^
*Azadirachta indica*	1.18 ± 0.06^a^	0.12 ± 0.01^b^	23.38 ± 3.53^b^	45.00 ± 1.95^a^	23.80 ± 5.24^a^	30.16 ± 1.76^b^	0.52 ± 0.09^c^	19.74 ± 1.87^b^	189.17 ± 5.42^b^	38.23 ± 3.00^c^	368.98 ± 50.68^bc^	9.62 ± 0.63^a^
*Dalbergia sissoo*	1.23 ± 0.06^a^	0.07 ± 0.01^a^	22.44 ± 5.65^b^	46.80 ± 3.71^a^	35.63 ± 1.43^b^	44.03 ± 1.05^bc^	0.48 ± 0.12^b^	18.15 ± 4.63^b^	296.52 ± 47.61^c^	37.81 ± 0.94^c^	626.70 ± 55.54^d^	16.58 ± 1.56^b^
*Pongamia pinnata*	2.92 ± 0.08^b^	0.21 ± 0.04^c^	17.02 ± 2.63^a^	44.50 ± 3.07^a^	49.40 ± 2.08^c^	20.73 ± 1.55^a^	0.38 ± 0.03^a^	5.80 ± 0.75^a^	81.93 ± 21.68^a^	15.21 ± 0.63^a^	214.27 ± 48.21^a^	14.06 ± 2.93^b^
*Terminalia arjuna*	1.24 ± 0.05^a^	0.13 ± 0.01^b^	21.43 ± 0.99^b^	46.00 ± 0.99^a^	29.43 ± 3.57^a^	38.00 ± 2.00^b^	0.46 ± 0.02^b^	17.30 ± 1.50^b^	161.68 ± 13.31^b^	37.08 ± 1.13^c^	347.33 ± 30.10^bc^	9.37 ± 0.84^a^
**Exotic species**												
*Broussonetia papyrifera*	3.78 ± 0.13^c^	0.23 ± 0.02^b^	7.38 ± 0.85^a^	42.00 ± 0.79^a^	9.73 ± 1.10^a^	37.83 ± 9.87^c^	0.17 ± 0.02^a^	1.95 ± 0.25^a^	31.73 ± 5.70^a^	11.10 ± 0.18^a^	180.35 ± 21.36^a^	16.25 ± 2.13^a^
*Eucalyptus globulus*	1.13 ± 0.05^a^	0.09 ± 0.01^a^	24.63 ± 3.17^b^	47.00 ± 0.99^a^	17.80 ± 2.40^b^	32.03 ± 0.95^c^	0.52 ± 0.07^c^	21.80 ± 3.73^c^	255.65 ± 43.43^b^	41.45 ± 2.20^b^	485.96 ± 15.90^c^	11.73 ± 0.38^a^
*Grevillea robusta*	1.01 ± 0.01^a^	0.07 ± 0.01^a^	26.26 ± 3.02^b^	48.10 ± 0.71^a^	13.60 ± 0.45^ab^	14.36 ± 7.34^a^	0.54 ± 0.05^c^	25.92 ± 3.15^c^	373.61 ± 57.20^c^	47.48 ± 1.38^b^	682.81 ± 34.32^d^	14.3 ± 0.51^a^
*Lantana camara*	2.88 ± 0.63^b^	0.18 ± 0.01^a^	13.48 ± 1.38^c^	43.40 ± 0.47^a^	2.10 ± 0.10^c^	45.76 ± 1.64^d^	0.31 ± 0.02^b^	4.86 ± 1.31^b^	73.59 ± 10.94^d^	15.52 ± 3.24^a^	236.40 ± 15.15^b^	15.69 ± 3.41^a^
*Leucaena leucocephala*	2.97 ± 0.01^b^	0.19 ± 0.01^a^	14.80 ± 3.10^c^	43.00 ± 1.00^a^	8.63 ± 1.01^a^	29.23 ± 11.60^b^	0.34 ± 0.07^b^	4.98 ± 1.05^b^	78.07 ± 23.34^d^	14.46 ± 0.36^a^	223.70 ± 19.61^b^	15.47 ± 1.44^a^

L = Lignin, C = Carbon, N = Nitrogen, P = Phosphorus.

### Litter chemical quality among native and exotic species

**Figure 4:**
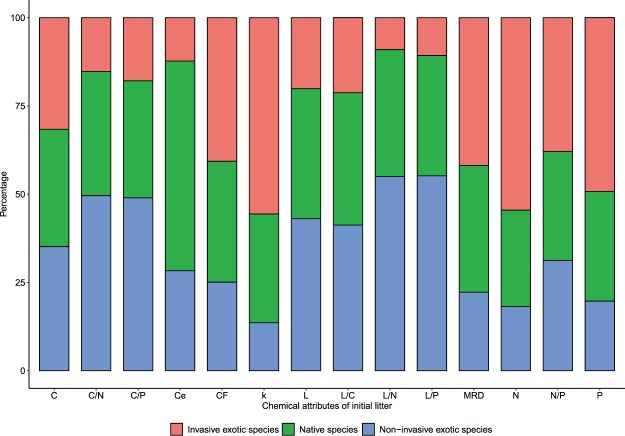
Stack bar showing the decomposability and chemical attributes of leaf litter among the Native species (n = 5), Invasive exotic species (n = 3) and Non-invasive exotic species (n = 2). The mean values of each parameter are taken as average among the species group and represented as percentage contribution to each parameter.

The variation of chemical attributes of the initial litter among native, invasive exotic and non-invasive exotic species are shown in Fig. 4. In contrast to our hypothesis, native and exotic species did not differ significantly in their initial litter chemistry (see Supplementary Table S2). All parameters of litter chemical characterisation in the present study showed non-significant relationships except cellulose content which was significantly (*t* = 3.313, *p* = 0.030) higher in native species as compared to the exotic species (see Supplementary Table S2). Litter cellulose, lignin to carbon ratio, lignin to phosphorus ratio and carbon to nitrogen ratio were significantly lower in invasive exotic species as compared to native species. None of the litter chemical parameters is found significantly different among non-invasive exotic species and native species. However, most of litter chemical parameters (except litter cellulose, crude fibre and nitrogen to phosphorus ratio) significantly differed between selected invasive and non-invasive exotic species. Litter nitrogen and phosphorus were significantly higher in invasive exotic species whereas lignin, carbon, lignin to carbon ratio, lignin to nitrogen ratio, lignin to phosphorus ratio, carbon to nitrogen ratio and carbon to phosphorus ratio were significantly lower in invasive exotic species as compared to non-invasive exotic species (see Supplementary Table S2). Analysis of variance (ANOVA) indicated significant differences due to species, origin and their interactions (species × origin) for litter chemistry parameters (Table 2). However, the carbon content of leaf litter was not significantly varied due to origin and species but significantly differed in their interaction (species × origin). Similarly, crude fibre content was insignificant due to origin but significantly varied due to the interaction of species and origin (species × origin) (Table 2).

### Correlation between decomposability traits and litter chemistry

**Figure 5:**
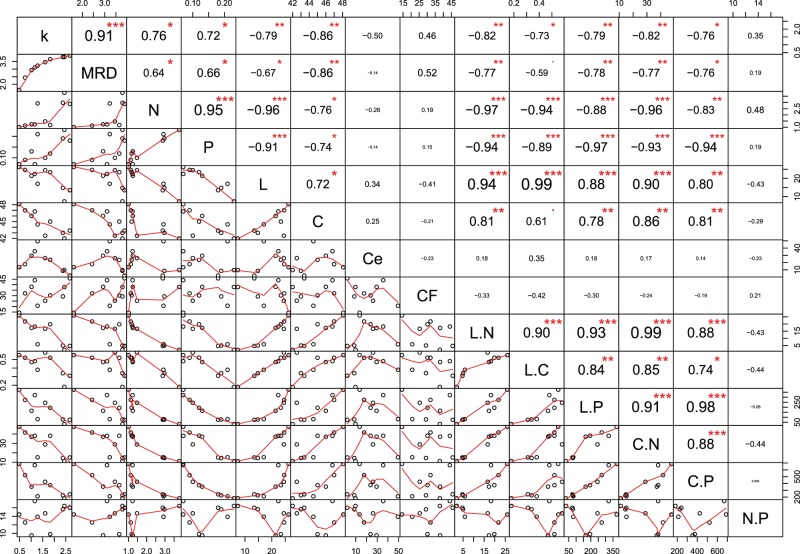
The plot shows the correlation between annual decay constant (*k*) and the chemical quality of the initial litter. The diagonal represents the distribution of each variable. Bivariate scatter plots with a fitted line are shown on the lower side of the diagonal while the value of Pearson’s correlation coefficient (R^2^) along with significance level indicated with stars are shown on above the diagonal. The significance level in terms of p-values 0.001, 0.01, 0.05 and 0.1 corresponds to “***”, “**”. “*” and “.”, respectively.

The decomposition rate of litter (in terms of *k* and MRD) has correlated with initial chemical quality of litter (except cellulose, crude fibre and nitrogen to phosphorus ratio) and the results are depicted in Fig. 5. The decomposition rate was negatively correlated with initial leaf litter lignin, carbon, lignin to nitrogen ratio, lignin to carbon ratio, lignin to phosphorus, carbon to nitrogen ratio and carbon to phosphorus ratio whereas it was positively correlated with litter nitrogen and phosphorus in the present study (Fig. 5). There is no significant correlation was found between decomposition rate and cellulose, crude fibre and nitrogen to phosphorus ratio. This is also advocated by the bivariate scatter plots of the decay constant (*k*) and litter chemistry parameters (see Supplementary Fig. S3). Litter carbon, lignin to nitrogen ratio and carbon to nitrogen ratio were the most significant predictors of the decomposition rate. Although nutrient contents are independent of each other, our results indicated significant correlations among some litter chemical attributes. Litter lignin was negatively correlated with nitrogen and phosphorus content whereas litter nitrogen and phosphorus were positively correlated (Fig. 5).

### Nitrogen and Phosphorus dynamics during decomposition

The nitrogen and phosphorus content of litter varied with time as decomposition progressed. The changes in litter nitrogen content with time did not follow any pattern among species, but in most cases, it first decreased and then increased (see Supplementary Fig. S5). Overall, the nitrogen content of litter increased with time for native and non-invasive exotic species whereas it decreased for invasive exotic species (see Supplementary Fig. S6). On the other hand, the phosphorus content of litter decreased with time during the decomposition for all the species (see Supplementary Fig. S7). However, the magnitude of this decrease was substantially higher for invasive exotic species and lower for non-invasive exotic species as compared to native species (see Supplementary Fig. S8).

## Discussion

As already mentioned, the present study aimed to compare the decomposability of leaf litter of selected native and exotic plant species. In contrast to our expectations, the present study did not find any significant difference in overall decomposition rates of selected native and exotic woody species, which is in line with earlier reports^26,27^. This suggests that the origin of species does not influence the rate of litter decomposition. However, we found that invasive exotic species decompose faster than native as well as non-invasive exotic species in the present study as well as previous studies^23–25,58,59^. Thus, it can be inferred that some exotic species, but certainly not all the species, decompose their litter faster than native species. Further, it also appears that different species have a variable effect on the decomposition but not all the species have an effect of the same magnitude. It may be possible that faster decomposition of litter may enhance the competitive ability of these plants in newer ranges by rapid availability of nutrients which can be utilised to produce higher biomass and reproductive growth. Further, the decomposition rate of exotic litter may also vary from non-invasive exotic to invasive exotic indicating that litter decomposition rate may be linked with the invasion success of some exotic species. It may be possible that decomposition rate may increase through successive stages of invasion: introduced, naturalisation, prior invasion and final invasion. Therefore, further studies should consider species effects and invasion stage of individual species to get insights into species and litter decomposition relationships.

As per our expectations, there are considerable differences in tissue chemistry of individual species that can be due to interspecific differences in functional attributes and nutrient use efficiency^47^. Although overall differences due to the origin of species (native vs exotic) were negligible and non-significant, invasive exotic species had exhibited relatively high foliar nitrogen and phosphorus content, and lower lignin content in the present study as well as previously reported^24,26,53,59^. The higher leaf litter quality (nitrogen and phosphorus) for *B*. *papyrifera* and *L*. *camara* are comparable with earlier studies^60,61^. On the other hand, the relatively higher nitrogen content of leaf litter of *A*. *lebbeck*, *D*. *sissoo* and *P*. *pinnata* among native species, can be attributed to nitrogen fixation by symbiotic *Rhizobia*. Other native and non-invasive exotic species may have poor assimilation efficiency to use and allocate nitrogen or phosphorus and therefore, they maintain a higher number of carbon-containing compounds such as lignin which may act as a defence to herbivores and other enemies. Interestingly, cellulose was significantly lower in exotic species as compared to native species, suggesting that native plants invest more in carbon-containing metabolites resulted in slower decomposability of native litter. Further, non-invasive exotic plants also have chemical constituents similar to native species indicating that they may face similar kind of environmental conditions and natural enemies.

The “enemy release (ER) hypothesis” for biological invasion postulates that uncontrolled growth of exotic species in non-native environments may be due to lower pressure from enemies such as predators and pathogens^18^. Thus, in cases where the ER hypothesis of biological invasions holds, it is presumed that the species especially plant will tend to invest less in defence and more in the construction of tissues which will maximise their competitive fitness (such as biomass and reproduction), if resources are limited. This is why invasive exotic plants may have higher leaf litter quality in terms of higher nutrient contents (C, N, P) and lower structural defence compounds (lignin and cellulose)^24,26,53,59–62^. This high leaf litter quality of invasive exotic plants suggests their high nutrient-resource use efficiency, which may increase their competing ability and therefore, fulfil of invasion success^61^. Further, it may be possible that invasive exotic plants tend to increase soil fertility, fasten nutrient cycling and modify their environment that is ideal for them as compared to other species^63^. Thus, in absence of natural enemies, the evolutionary selection will favour such genotypes with increased competitive abilities and low resource allocation to defence, this prediction is known as the “evolution of increased competitive ability (EICA) hypothesis”^64^. Although the evolution of invasive plants is evident, a little support for this hypothesis is available in the literature so far^11,65^. Surprisingly, our results seem to support this EICA hypothesis of biological invasion. Furthermore, the invasive plants are expected to have reduced quantitative (such as reducing palatability and digestibility) defences for ‘generalists’ as compared to qualitative defence (such as toxins) for ‘specialists’ due to higher construction costs for the production of quantitative defences. Therefore, a shift from quantitative to qualitative defences should increase the competitive ability of exotic plants via net gain in resources in newer ranges. This leads to the refinement of EICA and resulted in a relatively more supported^11^ hypothesis called as the “shifting defence (SD) hypothesis”^66^. Thus, our findings seem to advocate the SD hypothesis as indicated by a relatively lower amount of quantitative defence (lignin) in invasive plants as compared to native and non-invasive plants. The lower amount of quantitative defence in invasive plants also suggests that these plant face relatively lesser generalist enemies whereas native and non-invasive plant have a larger number of generalist enemies. Thus, indirectly suggesting the release of generalist enemies in the newer ranges. These hypotheses (EICA and SD) may be complemented by the much supported “phenotypic plasticity (PP) hypothesis” which posits that invasive species may have a higher degree of phenotypic plasticity as compared to non-invasive exotic or native species in their non-native ranges^11,67,68^. In addition to these, the phylogenetic relatedness of invading exotic species and that of native species in a newer habitat also affects the invasion success of a species in a habitat. In this context, the “Darwin’s naturalisation hypothesis” postulates that invasion success of exotic species tend to be higher in a new habitat that is poor in closely related (phylogenetic) native species^69^ whereas the “limiting similarity hypothesis” posits that greater difference in similarity (functional) between exotic and native species tends to facilitate the invasion success of exotic species^70^ and a fair amount of available evidence supports both the hypotheses^11^. However, the present study did not find significant evidence for these hypotheses possibly due to a smaller number of species considered.

Previous studies have shown that the litter quality in terms of its chemical composition is one among several factors, implicated for controlling the decomposition rates of the litter^26,71^. Litter chemistry influences the rate of litter decomposition primarily by controlling the activity of decomposer organisms because litter serve as the source of energy and nutrition to these organisms^40,58^. Litter carbon is an important source of energy to the decomposers, however, not all the carbon is available to them, because of structural carbon-containing compounds such as lignin and cellulose yield very little energy. Further, these compounds tend to increase rigidity which reduces palatability to decomposers and therefore, result in the slower rate of decomposition^40^. This agrees with the observed negative correlation with carbon content and lignin content of initial litter in the present study as well as several other studies^26,30,50–53^. Further, the significant positive correlation of leaf litter nitrogen content with decomposition rate is in agreement with earlier as well as recent reports^49,52–54^. This can be attributed to increased activity of associated microbial communities and soil-decomposer fauna with greater availability of nitrogen, which is an essential nutrient required for their growth, metabolism and reproduction^58^. Similarly, the positive correlation observed for initial phosphorus content is supported by earlier study^72^. This positive correlation may again reflect the increased activity of soil biota due to availability of another essential nutrient. Since structural carbon-containing compounds, such lignin and cellulose tend to slow down decomposition rate whereas essential nutrient such as nitrogen and phosphorus tend to promote the litter decomposition, therefore the ratio of lignin to nitrogen (L/N)^52,53^ and carbon to nitrogen (C/N)^27,48,49^ have been suggested as key predictors of decomposition rates. These complementary effects of nitrogen and lignin or carbon to enhance the litter quality were also observed in the present study as evidenced by a good correlation of L/N and C/N with annual decay rate constant. Thus, chemical traits like L/N and C/N ratios have pronounced control over the rate of decomposition probably complemented by environmental and habitat effects.

Although the foliar nutrient contents are generally thought to be independent of each other, we have observed correlations among the litter chemical composition in the present study. The strong positive correlation of litter nitrogen and phosphorus indicate that increased foliar nitrogen may promote fungal diversity and their activity which results in higher phosphorus availability to the plants^73^. Similarly, a strong negative correlation of lignin content with both nitrogen and phosphorus suggests that whenever plants have the availability of nitrogen and phosphorus, they tend to invest less on carbon-containing secondary metabolites like lignin.

The litter chemical composition often changes as the decomposition progresses due to bio-chemical activities of microbes and their enzymes. It has been acknowledged that both nitrogen and phosphorus content of litter increases during decomposition, however, the reasons for this increase are not clear. The nitrogen content seemed to increase with time for native and non-invasive exotic species but not for the invasive exotic species at least for the selected woody species. In the present study, litter nitrogen content seems to first decrease possibly due to some amount of leaching and then increases which may be contributed by microbial biomass nitrogen. The overall decrease in nitrogen for invasive species might be due to the formation of leachable nitrogen compounds. It may also be possible that whenever the decomposer organisms are not able to derive sufficient amount of nitrogen from litter, they tend to acquire nitrogen from other non-litter sources (such as feeding on other organisms) in the soil. This may explain the general decrease in nitrogen content when the initial nitrogen in a litter is high as in case of invasive exotic plants. However, the overall decreasing pattern of litter phosphorus content during decomposition may be due to its conversion into leachable form. Also, the fungal group of decomposers may convert the litter phosphorus into the available form which might be readily used by plants and other microbes.

However, the present study has some limitations as it considered only leaf litter, litter from other non-leaf parts may potentially influence the rate of decomposition in terrestrial ecosystems. Further, the period for the study was limited (just a single year) that can be misleading to arrive at a concrete conclusion. This is because leaching and degradation of high-quality compounds are higher during the initial phases. Another important shortcoming is that the litterbag method may overestimate or underestimate decomposition rate because it prevents the fragmentation of litter and only estimates the combined effect of leaching and catabolism by mass loss. Therefore, further studies should also concern the effects of fragmentation while estimating the litter decomposition rates with other kinds of recent techniques whosever greater accuracy may reflect satisfactory results. Furthermore, since our study did not indicate any significant differences due to the origin of species, it might be possible that the litterbag method can be inefficient to capture the effects of the origin of species. Therefore, we suggest to include different techniques (such as transplant experiments, soil respiration, litter bed etc.) to study the effects due to the origin of the species. We recommend future research to include non-leaf litter and expand the study time scale as well as the number of pairs of exotic-native species to reduce biases.

Overall, our study revealed that the rate of litter decomposition in urban environments is highly influenced by initial litter chemical quality rather than the origin of species. However, the enhanced decomposition rate in case of invasive exotic plants can be attributed to their higher leaf litter quality, which is preferred by decomposers^24,26,59,62^. On the other hand, the relative slow decomposition rate of native and non-invasive species can be attributed to their low-quality litter with high lignin which provides rigidity to leaves and makes them less palatable to decomposers. Thus, the better quality of litter^51,74^ promotes the activity of decomposers which eventually results in faster decomposition of litter^23–25^. Moreover, this fast decomposition results in rapid nutrient cycling, which tends to increase soil fertility by the faster release of nutrients in soil^75^. Thus, higher nutrient availability in soil probably promotes growth and reproduction of invasive exotic species by increasing resource use efficiency which enables them to outcompete the native species. This facilitation due to rapid nutrient cycling along with climatic benefits is supposed to increase plant invasion, which can alter major ecological processes and services including nutrient cycling, especially the carbon and nitrogen cycles.

## Conclusions

The present study demonstrated that the decomposition rate of leaf litter did not significantly differ between exotic and native species in the same urban environment. However, the litter of invasive exotic species decomposed rapidly, whereas, the litter of non-invasive was slowly decomposed as compared to native species. Further, the rate of decomposition is significantly correlated with initial chemical quality such as carbon to nitrogen ratio and lignin to nitrogen ratio of the leaf litter. Since litter chemical quality increased from non-invasive exotic to invasive exotic species, it suggests that invasive exotic plants face lesser enemies in the newer ranges and therefore tend to invest less in defence, and that results in the production of high-quality litter. This high-quality litter decomposes rapidly and increase nutrient availability in the soils, which in turn favour their growth and reproduction through a positive feedback mechanism. Thus, in conclusion, our results seem to support the “evolution of increased competitive ability (EICA)” and “shifting defence (SD)” hypotheses of biological invasions in urban ecosystems. Further, it is reasonably elicited that the rate of litter decomposition is greatly influenced by litter quality rather than the origin of species. It will be more interesting, if the same results can be obtained in future investigations, considering both the leaf and non-leaf litter from different stages of invasion like introduction, naturalisation, prior invasion and final invasion.

## Materials and Methods

### Study site and Climate

City Chandigarh covers an area of about 114 km^2^ and located near the foothills of the Siwalik range. The city lies in the north-western part of India (30° 44ʹ14” N latitude and 76° 47ʹ14” E longitudes) at an average elevation of 321 m. The present study was conducted on leaf litter of selected woody species which are collected from forest sites located in and around Chandigarh city. The collected leaf litter was then incubated for decomposition on the soil surface of a fenced enclosure at P. N. Mehra Botanical Garden of Panjab University, Chandigarh, which offered us common garden conditions for both types of species. Since this site has both exotic and native plant species grown together, it offered a relatively unbiased study site regarding home-field advantage for litter decomposition. This area of Siwaliks is dominated by alluvial sediments chiefly consisting of loose sand and gravel inserted with clay horizons. Soil is clay loam or silt loam and underlying rocks are soft sandstones that are prone to erosion. The alkali soils of the region are low in organic matter and deficient in nutrients, probably due to minimal leaf litter. The physico-chemical properties of the soils of selected study sites are summarised in Supplementary Table S3.

Chandigarh has a humid subtropical climate characterised by a seasonal variation; very hot summer, mild winter, unreliable rainfalls but evaporation usually exceeds precipitation. In the spring season, temperature varies between 13 °C to 20 °C (maximum) and 5 °C to 12 °C (minimum). The temperature in autumn usually remains between 10 °C to 22 °C but it may rise to a maximum of 30 °C and may fall to a minimum of around 6 °C. The temperature of summer is long, attaining maximum temperature 44 °C during the mid-June and it generally remains between 40 °C and 42 °C. The average temperature in the winter remains at 5 °C to 14 °C (maximum) and −1 °C to 5 °C (minimum). The rain usually comes from the west during winters and it is usually a persistent rain for 2–3 days with sometimes hailstorms. During monsoon, Chandigarh receives moderate to heavy rainfall and rain usually bearing monsoon winds.

### Selection of Plant species

To compare decomposability traits, we have randomly selected five native and five exotic plant species that were fairly abundant within as well as the surrounding area of the city. The origin (native or exotic) of the selected species were confirmed by comparing with literature available from CABI-Invasive Species Compendium^76^. The selected native species were *Albizia lebbeck* Benth., *Azadirachta indica* A. Juss., *Dalbergia sissoo* Roxb., *Pongamia pinnata* L. and *Terminalia arjuna* Roxb. On the other hand, the following five exotic species were selected: *Broussonetia papyrifera* (L.) L’Hér. ex Vent., *Grevillea robusta* A. Cunn., *Eucalyptus globulus* Labill., *Lantana camara* L. and *Leucaena leucocephala* Benth. The origin and introduction of exotic species has been briefly described as follows: *Broussonetia papyrifera* (L.) L’Hér. ex Vent. (Moraceae) is a native tree of eastern Asia, which was introduced to India as an avenue tree in 1880 at Saharanpur^77^. This tree has already been predicted to become common in sub-Himalayan tract^77^ and now it has been recorded as one of the fast-growing invaders in the region^78^. *Eucalyptus globulus* Labill. (Myrtaceae) is a fast-growing large tree is native to Australia and is propagated from seeds. It was first planted around 1782 by Tippu Sultan, in his palace garden on Nandi hills (near Bangalore) for ornamental purposes. Later, in the 1850s the East India Company planted it in Lucknow, Saharanpur, Dehradun and Lahore for commercial pulpwood fibre and timber^79^. *Grevillea robusta* A Cunn. (Proteaceae) is an evergreen native tree of eastern-coastal Australia, growing in riverine, subtropical and dry rainforest environments. It is planted in India during the mid to late 1800s probably as a shade-providing tree for tea and coffee plantations. Later it was introduced to northwest regions for avenue purposes^80^. *Lantana camara* L. (Verbenaceae) is an evergreen shrub with the native range in Central and South America. In India, it is introduced as early as 1807 for its beautiful ornamental flowers^81^. *Leucaena leucocephala* Benth. (Fabaceae) is a short scrubby tree which is native to tropical America. It was introduced to the Western Ghats in India in the late 1800s and is now reported to be expanding rapidly in all habitats^81^. Among exotic species, three species (*Broussonetia papyrifera* (L.) L’Hér. ex Vent., *Lantana camara* L. and *Leucaena leucocephala* Benth.) has become terrible invaders and considered as invasive species across the country^78^. Thus, all the selected woody species have been grouped as native species (*A*. *lebbeck*, *A*. *indica*, *D*. *sissoo*, *P*. *pinnata* and *T*. *arjuna*), invasive exotic species (*B*. *papyrifera*, *L*. *camara* and *L*. *leucocephala*) and non-invasive exotic species (*E*. *globulus* and *G*. *robusta*). After species grouping, they were analysed for phylogenetic relationships (based on Zanne *et al.*, 2014)^82^ and it was found that exotic species were significantly distantly-related whereas the native species were closely related, though not significantly (see Supplementary Table S4 and Fig. S4).

### Leaf litter collection and analyses of litter chemistry

For litter collection, mature healthy trees were identified and freshly fallen leaves of each species were collected. The age of selected native species for litter collection ranged from about 30–35 years while exotic species were about 20–30 years old. The initial leaf litter chemical quality was evaluated in terms of total nitrogen (N), total phosphorus (P), cellulose (Ce), lignin (L) and organic carbon content (C) using previously described standard methods^83–85^. The detailed bio-chemical methods can be found as Supplementary Bio-chemical Methods.

### Experimental design and litter bag experiment

For litter decomposition, 21 nylon net litter bags (1 mm mesh size) containing 4–5 g of air-dried leaf litter of each species were placed separately into three plots (1.0 m × 1.0 m size) on the soil surface of the experimental site in February. Therefore, a total of 210 litterbags of the total selected species (exotic and native) were placed in thirty plots of the same size. The experimental site was the fenced enclosure in P. N. Mehra Botanical Garden of Panjab University, Chandigarh. The dry weight of the litter was determined from the collected samples of litter stock. Three litterbags were recovered from each species after every 60 days on the pre-determined sampling dates. The recovered litter was air-dried, adhering soil particles were carefully brushed off, and then oven-dried at 80 °C and weighed using an electronic balance.

### Quantification of decomposition rates

For quantification of decomposition rates, we have evaluated 16 proposed models (see Supplementary Table S5) and selected the best model using the Akaike’s Information Criterion (AIC) and coefficient-of-determination (R^2^). In the present investigation, decomposition rate constant (*k*) calculated using a single exponential model that assumes a constant fraction of litter mass lost per unit time^86^.

$${W}_{t}={W}_{0}\,{e}^{-kt}\,or\,k=\frac{\mathrm{ln}[{W}_{0}]-\,\mathrm{ln}[{W}_{t}]}{t}$$

where *W*_*t*_ = Weight at time *t* and *W*_*0*_ = Initial weight of litter. The Mean relative decomposition rate (MRD) was calculated by using the formula:

$$MRD\,(g\,{g}^{-1}da{y}^{-1})=\frac{\mathrm{ln}({W}_{1}-{W}_{0})}{{t}_{1}-{t}_{0}}$$

where W_0_ = Weight of litter present at time *t*_*0*_, W_1_ = Weight of litter at time *t*_*1*_, and *t*_*1*_ – *t*_*0*_ = Sampling interval (days). Time taken for 50% decomposition and time for 95% was calculated using the following formula:

$${t}_{50}=\frac{0.693}{k};\,{t}_{95}=\frac{3}{k}$$

where *k* is decay constant.

Further, all the primary data and calculations are available as Supplementary Dataset 1.

### Statistical analyses

Data were subjected to a general linear model (GLM) for analysis of variance (ANOVA) using SPSS-PC statistical software (SPSS version 17.0, 2007). Correlations were carried out using the package *‘Performance Analytics’*^87^ and the phylogenetic relationships were analysed using the package ‘*picante’*^88^ in *R* programming language^89^. The weight loss data were fitted with a single exponential decay model using Sigma Plot version 14.0, from Systat Software, Inc. Other statistical analyses and calculations were performed using Microsoft Excel, which is also available as Supplementary Dataset 1.

## Supplementary information

Supplementary Information.

Dataset 1.

## Data Availability

All data generated or analysed during this study are included in this published article (and its Supplementary Information files i.e. Supplementary Dataset 1 and Supplementary Information).
