# Supplementary material for: Comparative litter decomposability traits of selected native and exotic woody species from an urban environment of north-western Siwalik region, India
Source: Sci Rep. 2020 May 12;10:7888. doi: 10.1038/s41598-020-64576-2 (PMC7217892; doi:10.1038/s41598-020-64576-2)
Supplement: Supplementary file 1 — Supplementary Information. [file 41598_2020_64576_MOESM1_ESM.pdf]

Comparative litter decomposability traits of selected native and exotic woody species from an urban environment of north-western Siwalik region, India

Meenu Patil, Abhishek Kumar, Pardeep Kumar, Navneet Kaur Cheema, Rupinder Kaur, Ramchand Bhatti and A. N. Singh*

Department of Botany, Panjab University, Chandigarh-160014, INDIA
*Corresponding author: ansingh@pu.ac.in; dranand1212@gmail.com

Table S1 The results of student's *t*-test to evaluate the significance of differences in litter decomposability parameters among native (n = 5), exotic (n = 5), invasive exotic (n = 3) and non-invasive exotic (n = 2) species used for decomposition study.

**Parameter**	**Native vs Exotic**		**Native vs Invasive exotic**		**Native vs Non-invasive exotic**		**Invasive exotic vs Non-invasive exotic**	
	t-value	*p*-value	*t*-value	*p*-value	*t*-value	*p*-value	*t*-value	*p*-value
k	-0.787	0.475	-5.320	0.002	2.979	0.031	12.398	0.001
MRD	0.396	0.712	-3.183	0.019	4.101	0.009	5.811	0.010
T_{50}	-0.553	0.610	3.363	0.015	5.689	0.002	-4.931	0.016
T_{95}	-0.552	0.611	3.346	0.016	-4.178	0.009	-4.925	0.016

Table S2 The results of student's t-test to evaluate the significance of differences in initial litter chemical quality among native (n = 5), exotic (n = 5), invasive exotic (n = 3) and non-invasive exotic (n = 2) species used for decomposition study.

**Parameters**	**Native vs Exotic**		**Native vs Invasive exotic**		**Native vs Non-invasive exotic**		**Invasive exotic vs Non-invasive exotic**	
	t-value	*p*-value	*t*-value	*p*-value	*t*-value	*p*-value	*t*-value	*p*-value
Nitrogen	-1.41	0.23	-	-	-	-	-5.75	0.01
Phosphorus	-0.84	0.44	-2.24	0.07	1.16	0.30	5.692	0.01
Lignin	1.21	0.29	4.12	0.06	-1.68	0.15	-4.50	0.02
Cellulose	3.31	0.03	4.12	0.00	2.23	0.07	-2.57	0.08
Crude fibre	-0.02	0.98	-0.90	0.40	1.00	0.36	1.60	0.20
Total carbon	0.29	0.78	2.11	0.08	-2.05	0.09	-7.03	0.00
L/C	-0.96	0.38	3.66	0.01	-0.90	0.40	-3.77	0.03
L/N	0.95	0.39	-	-	-	-	-9.97	0.00
L/P	0.65	0.55	2.68	0.03	-1.76	0.14	-5.29	0.01
C/N	0.88	0.42	2.99	0.02	-1.76	0.14	-10.87	0.00
C/P	0.51	0.63	2.01	0.09	-1.53	0.19	-4.84	0.02
N/P	-1.28	0.26	-1.54	0.17	-0.07	0.95	2.78	0.07

L = Lignin, C = Carbon, N = Nitrogen, P = Phosphorus, "-" = student's *t*-test was not conducted due to failure of normality test (Shapiro-Wilk test)

Table S3 Initial soil physico-chemical properties of selected study sites.

Soil parameters	Garden Site	Forest site
pH	7.36 – 7.45	7.13 – 7.68
Moisture content (%)	18 – 21	22 – 27
Water Holding Capacity (%)	35 – 37	34 – 36
Bulk density (g/cm^3)	1.13 – 1.15	1.13 – 1.17
Soil Organic Carbon (g/Kg dry soil)	18.10 – 19.20	4.90 – 5.10
Microbial Biomass Carbon (µg/g dry soil)	390.00 – 435.00	330.00 – 360.00
Mineral-N (µg/g dry soil)	40.50 – 45.30	32.05 – 34.00
Available-P_i (µg/g dry soil)	20.40 – 22.56	15.20 – 16.45
Total Kjeldahl Nitrogen (g/Kg dry soil)	1.32 – 1.35	1.10 – 1.14

Pi = Inorganic Phosphorus

Table S4 Phylogenetic relationships (based on Zanne et al., 2014) between exotic and native species evaluated using the package "*picante*" in *R* programming language.

**Phylogenetic parameter**	**Native**	**Exotic**	**Invasive**	**Non-invasive**
Faith's Phylogenetic Diversity (PD)	795.575	920.890	666.454	577.194
Phylogenetic Species Variability (PSV)	0.230	0.283	0.261	0.311
Phylogenetic Species Clustering (PSC)	0.185	0.267	0.252	0.311
Mean Pairwise Distance (MPD)¶	-1.001	1.306*	0.224	0.961
Mean Nearest Taxon Distance (MNTD)¶	-0.532	1.468*	0.417	0.996

¶= using the null model "taxa.labels" with 999 randomisations; *= significance level p > 0.95

Table S5 A list of models (y ~ t) which were evaluated using the *nlm* function (with Gaussian-Newton algorithm) in *R* programming language for the present study. Models were evaluated on the basis of Akaike's Information Criterion (AIC) and coefficient of determination (R^2).

Model Code	Models	Equation ($y = M_t/M_0$)	AIC†	$R^{2\dagger}$	AIC#	$R^{2\#}$
M1	Linear¶	$y = a + bt$	135.24	0.948	1777.81	0.680
M2	Quadratic	$y = a + bt + ct^2$	116.65	0.981	1758.55	0.713
M3	Cubic	$y = a + bt + ct^2 + dt^3$	118.65	0.981	1758.77	0.715
M4	Single Exponential (1)	$y = a\, e^{-t}$	229.84	-3.914	2242.84	-1.942
**M5**	**Single Exponential (2)**	$\boldsymbol{y = a\, e^{-kt}}$	**115.05**	**0.981**	**1755.38**	**0.714**
M6	Single Exponential (3)	$y = a\,(1 - e^{-kt})$	135.24	0.950	1777.81	0.682
M7	Single Exponential (4)	$y = a + e^{-kt}$	117.05	0.981	1757.20	0.714
M8	Log-linear¶	$\log y = \log a - kt$	-46.29	0.969	282.74	0.578
M9	Logarithmic¶	$y = a + b \log t$	1833.66	0.583	1833.66	0.583
M10	Power (1)	$y = t^{-a}$	228.07	-3.517	2252.29	-2.077
M11	Power (2)	$y = a\, t^{-b}$	1860.33	-6.174	1860.33	0.528
M12	Michaelis-Menten	$y = \frac{a\,t}{b + t} + c$	194.19	0.256	1756.83	0.715
M13	Hyperbola (1)	$y = \frac{a\,b}{b + t}$	131.54	0.958	1764.55	0.701
M14	Asymptotic (1)	$y = a\,(1 - e^{-kt/a})$	135.24	0.950	1777.81	0.682
M15	Double Exponential	$y = a\, e^{-k_1 t} + b\, e^{-k_2 t}$	-	-	1758.85	0.715
M16	Asymptotic (2)	$y = a + (y_0 - a)\, e^{-e^{k} t}$	117.05	0.981	1757.20	0.714

¶linear models tested with function *lm* in *R* programming language; †models tested for *Dalbergia sissoo* data; #models tested for pooled data (all species). The symbols used are as follows- *y* is the fraction of mass remaining at any particular time *t*; y0 is the value of y at *t* = 0; *k* is the decomposition rate constant, *k1* is decomposition rate constant for faster fraction whereas *k2* is the decomposition rate constant for slower fraction; *a*, *b* & *c* are the parameters for developing the models.

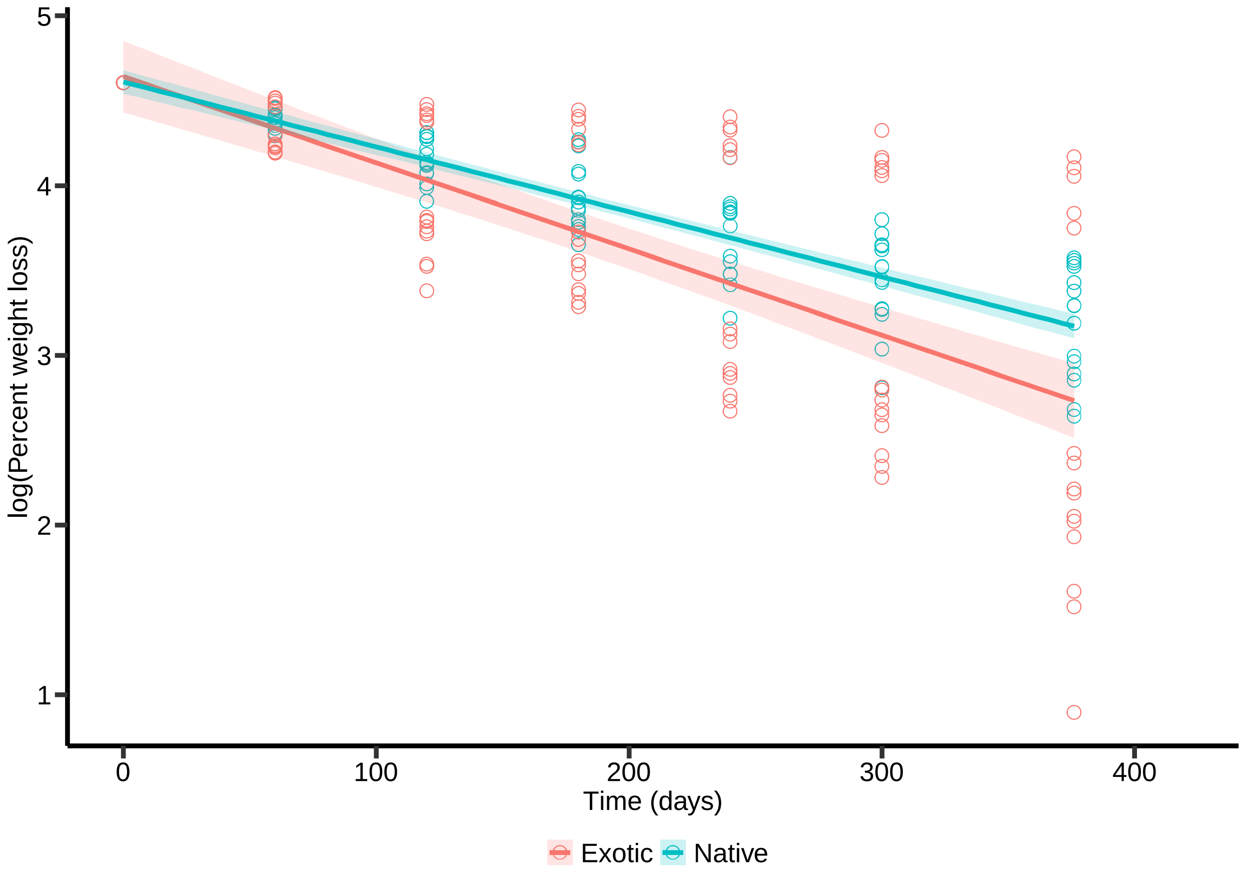

Figure S1 Decomposition rates of native and exotic species not differed significantly in the urban environment.

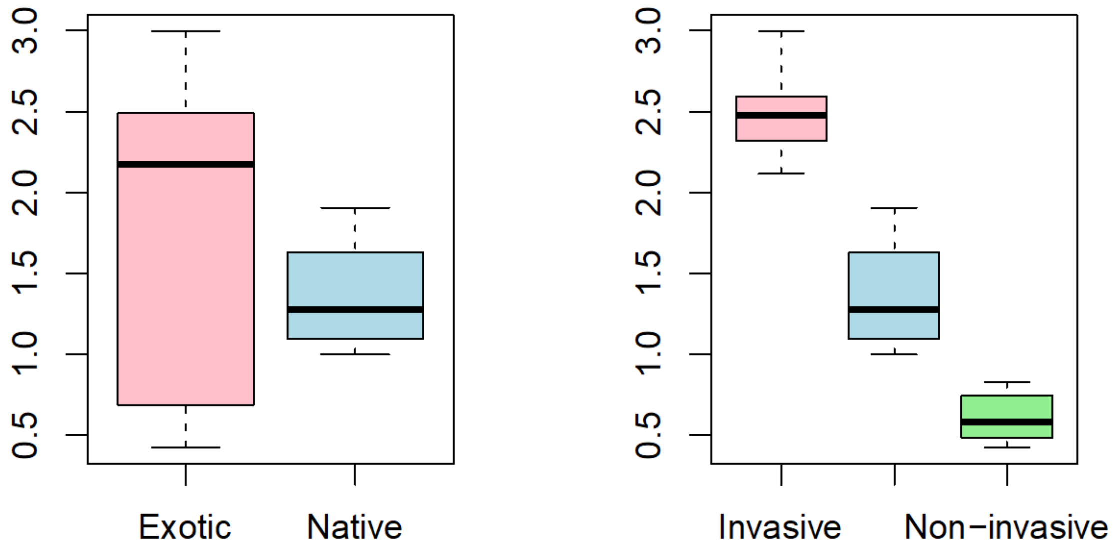

Figure S2 Decomposition rate of exotic species was highly variable as compared to native species. Invasive species had substantially higher decomposition rate, whereas, non-invasive species exhibited a lower rate as compared to the native species.

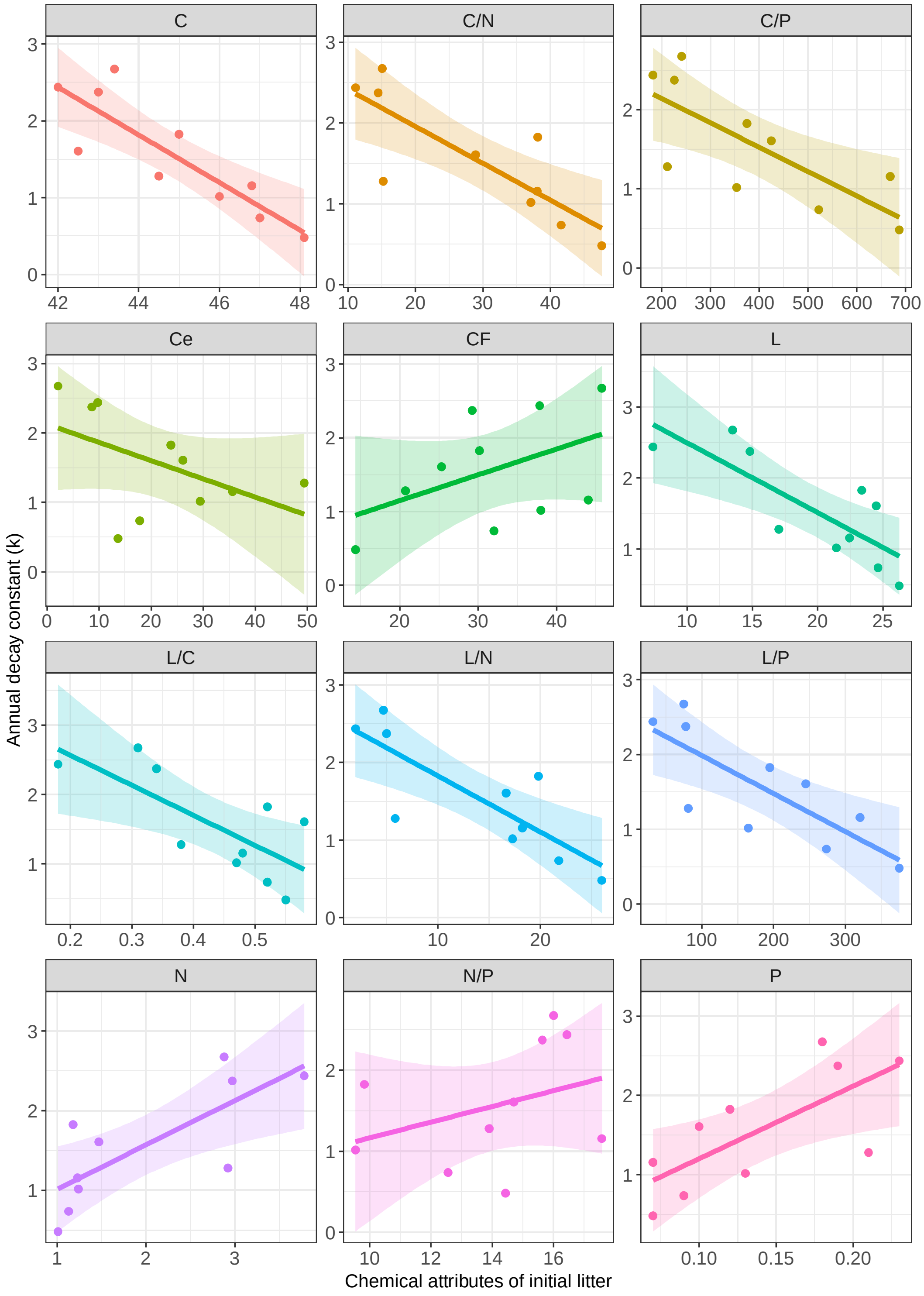

Figure S3 Bivariate scatter plots with a linear smooth line to depict the relationship between annual decay constant (*k*) and chemical attributes of initial leaf litter.

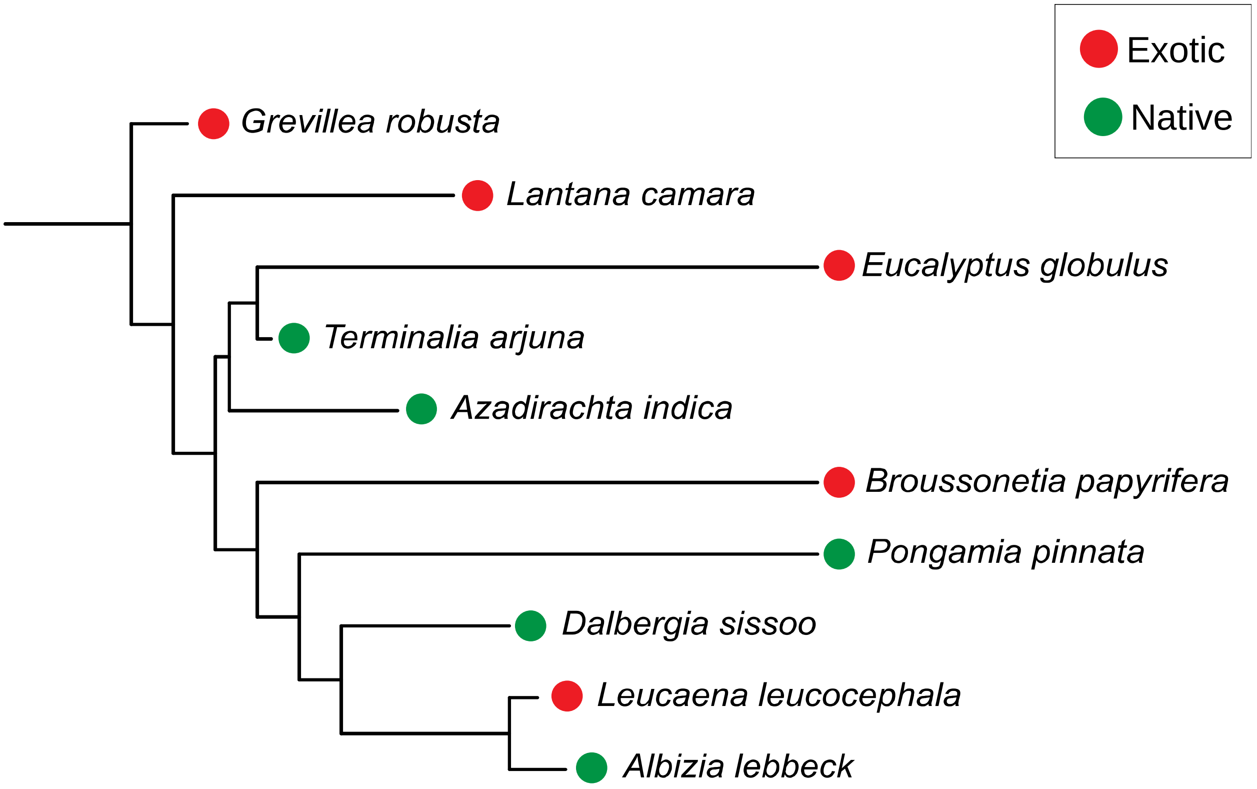

Figure S4 Phylogenetic relationships between the native and exotic species selected for the present study (based on the Zanne et al., 2014).

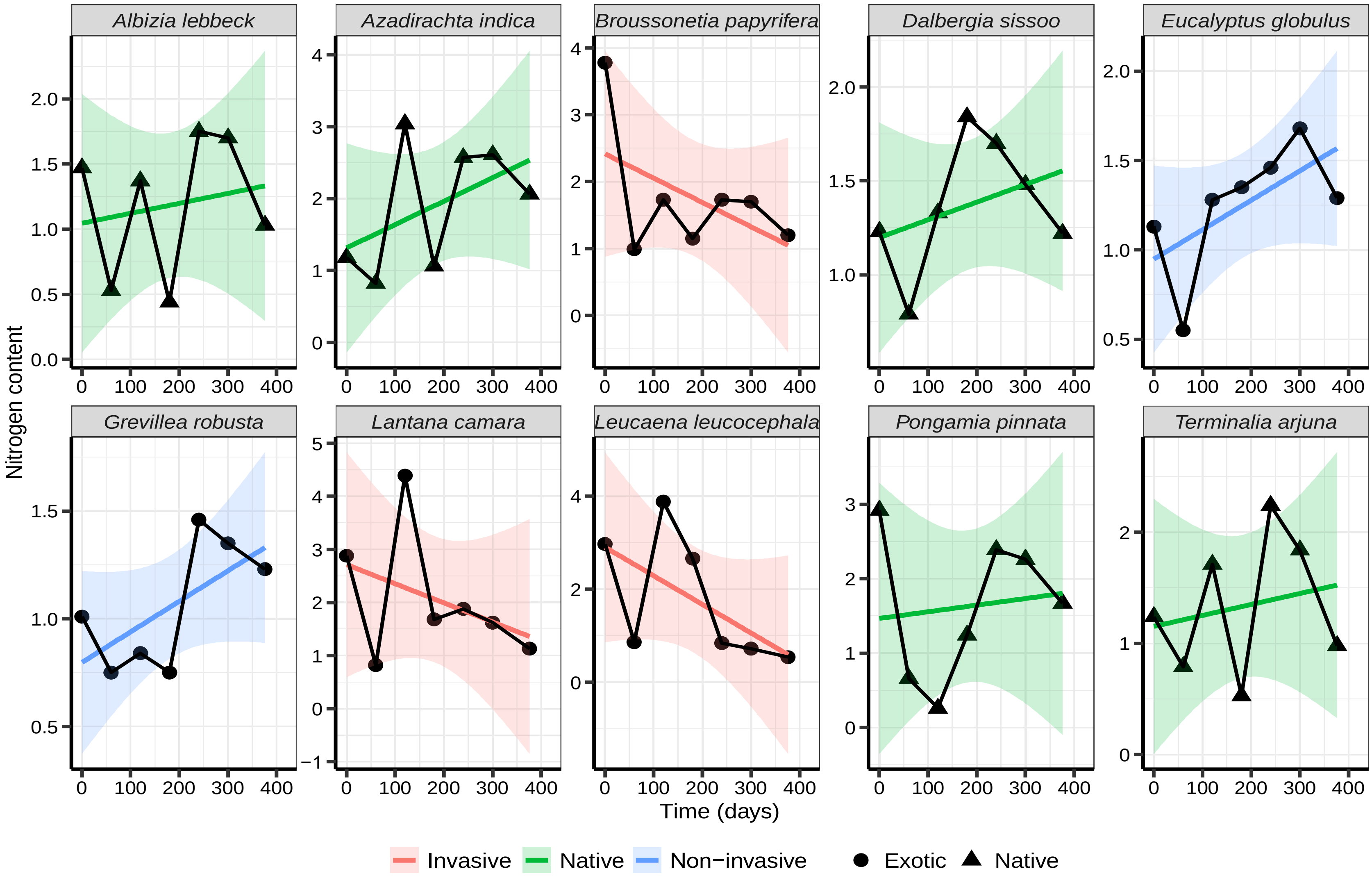

Figure S5 Nitrogen content of litter for different species varied with time during the decomposition.

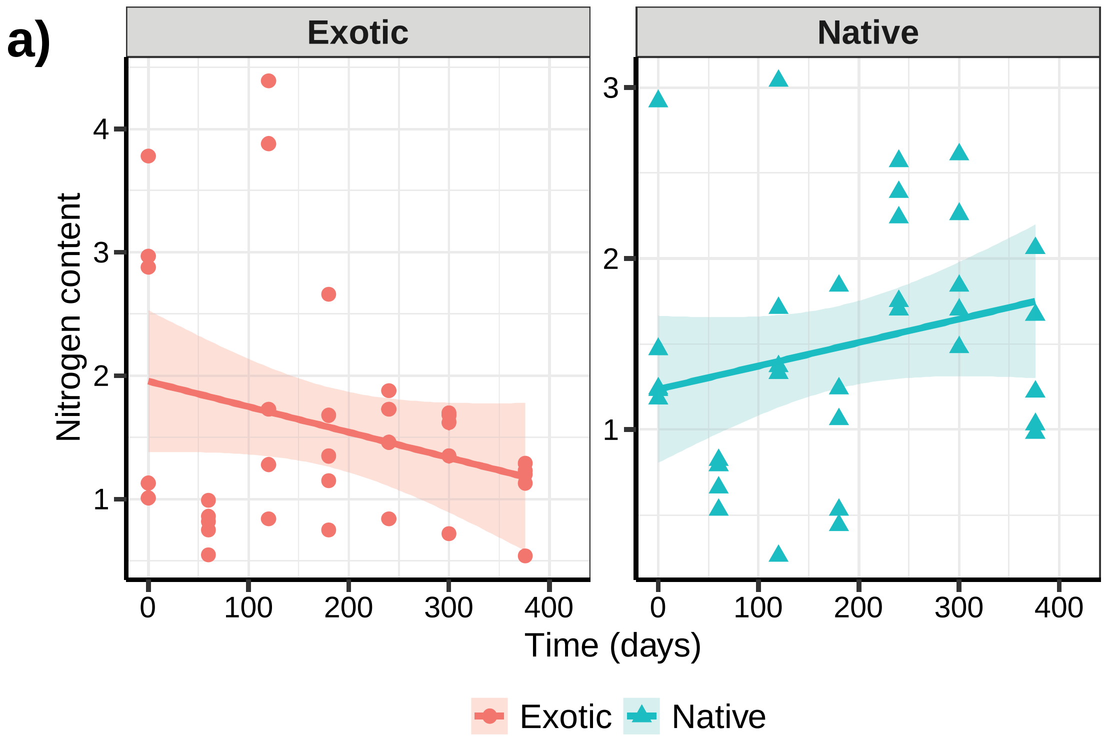

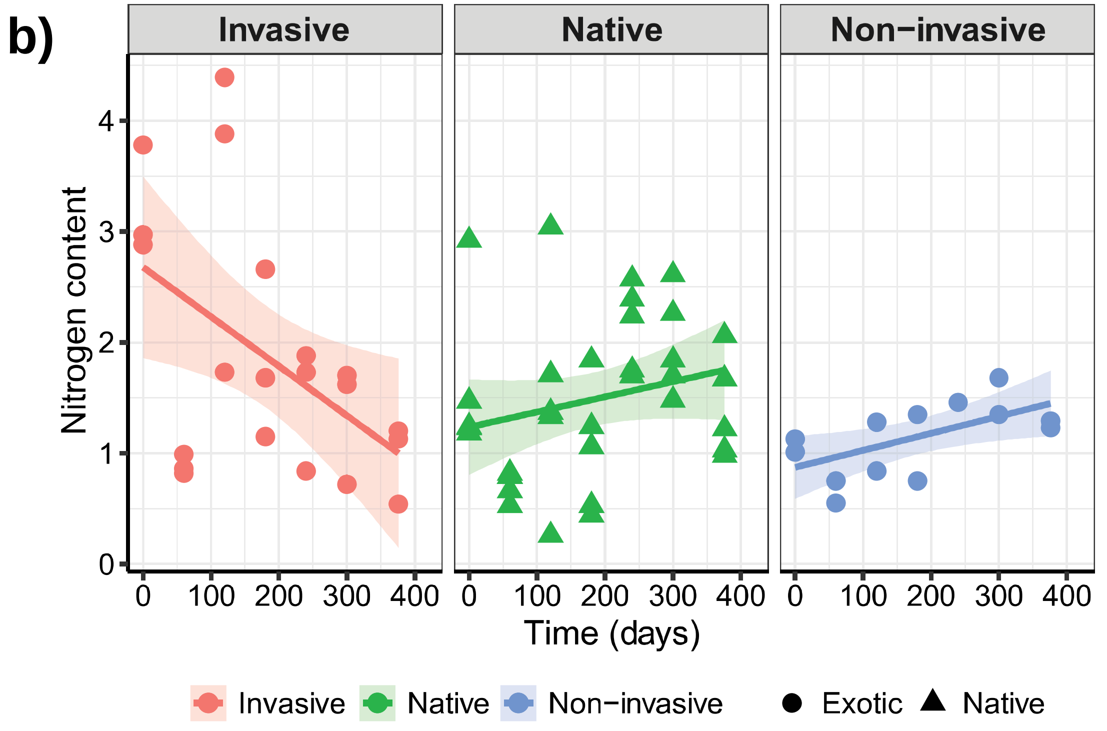

Figure S6 Nitrogen content of litter increased for native and non-invasive exotic species whereas decreased for invasive species during the decomposition.

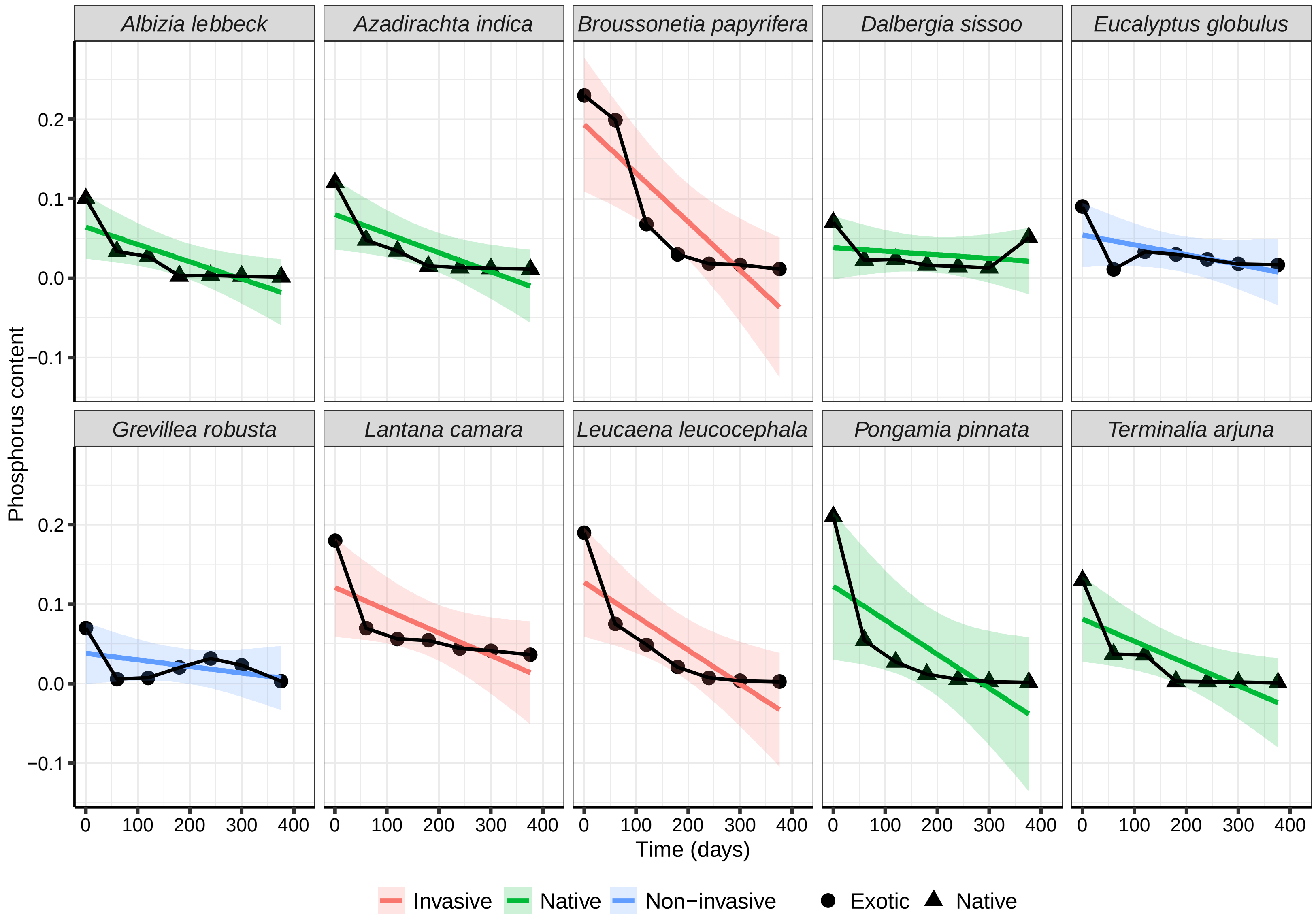

Figure S7 Phosphorus content of litter for different species decreased with time during the decomposition.

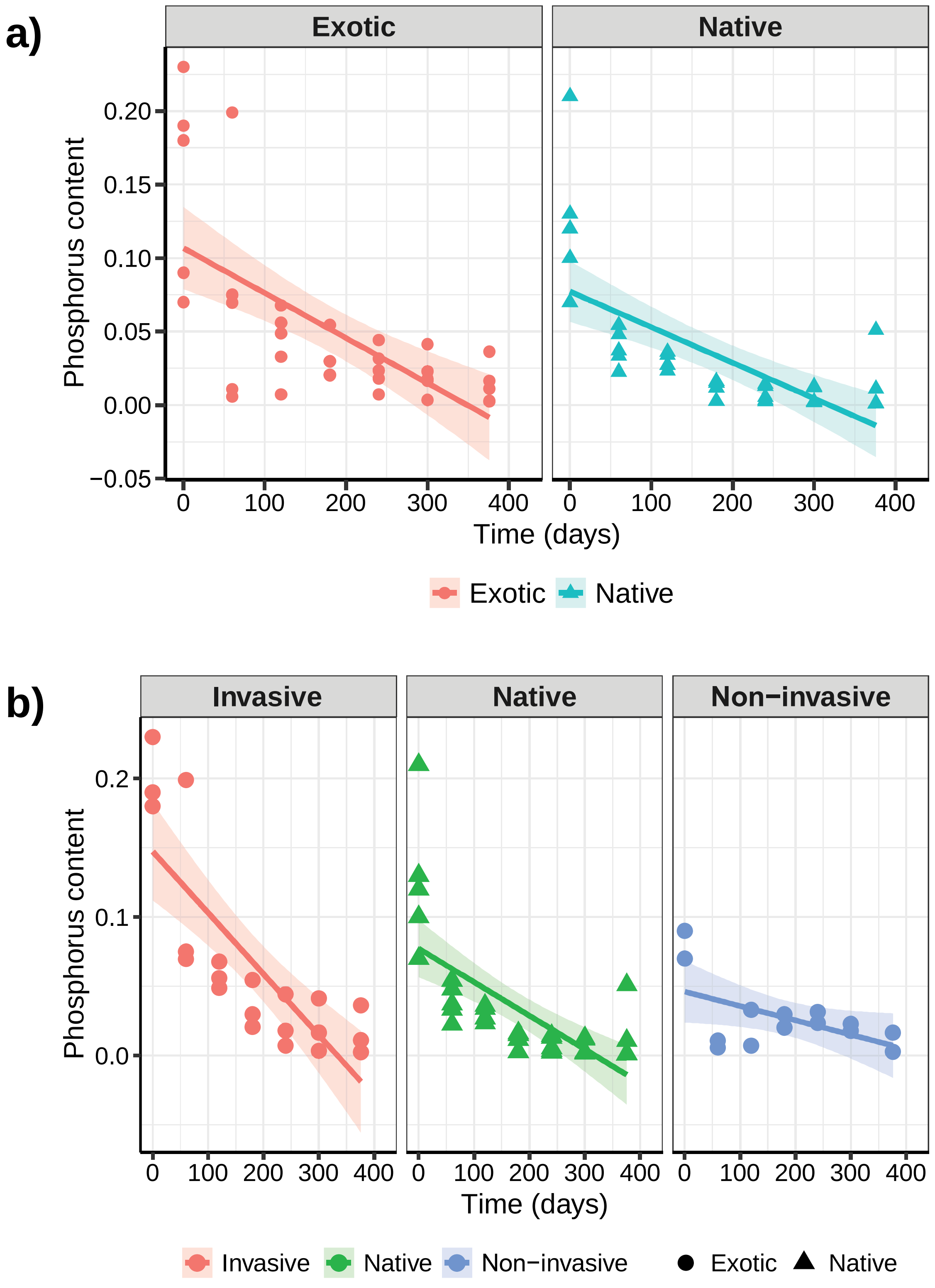

Figure S8 Phosphorus content of litter was decreased for exotic and native species with similar rates, but the decrease was higher for invasive and lower for non-invasive as compared to the native species.

Bio-chemical Methods

Digestion of samples

The freshly fallen senesced leaves of each species were collected and brought laboratory for processing; first air-dried and then dried in oven at 80 °C. Then, about 0.3 g (this weight was recorded as W) of oven dried (80 °C) litter was ground (0.25 mm, 60 mesh) into labelled, dry and clean digestion tube. Then, 4.4 ml of digestion mixture (mixture of 0.21 g Se powder and 7 g lithium sulphate to 175 ml 30% H_2O_2 and 210 ml conc. H_2SO_4) was added to each tube and also to the two reagent blanks prepared for each batch of samples. These mixtures (samples and blanks both) were digested in block digester (KELPlus-12) at 360 °C for 2 hours and then digestion tube kept in stand for allowed to cool. Then, about 25 ml of distilled water was added and mix well until no more sediment dissolves and then, allowed to cool further. After this, distilled water was further added to raise the total volume up to 50 ml and mixed well. It was allowed to settle so that a clear solution could be taken from the top of the tube for analysis. The standards were prepared with addition of 2.5 ml of digested digestion blanks together with samples

Total nitrogen content

Steam distillation apparatus was set up and steam was passed through the apparatus for 30 min. Steam blank was checked by collecting 50 ml distillate and titrating with N/70. An aliquot of about 5 ml from digested sample solution was transferred to the reaction chamber of the distillation unit and 10 ml of 40% NaOH was added to it. Then, this aliquot was steam distilled immediately into the mixture of 5 ml of 1% boric acid and 4 drops of the indicator. Distillation was continued for 2 minutes from the time the indicator turned green. The distillate was removed and titrated with N/70 HCl till the end point was being reached when the indicator changes from green through grey to a definite pink. Amount of standard HCl required was noted. A blank determination was run by digesting reagent blanks in place of the sample and distilling as above and titrating with N/70 HCl. The volume of N/70 HCl is corrected by subtracting the volume required (in ml) for the blank (usually 0.05 ml) from the micro-burette reading for end point. Total nitrogen was calculated using the formula:

$$\%N\ of\ Sample = \frac{Corrected\ volume\ of\ \frac{N}{70}\ HCl \times 0.2}{Weight\ of\ Sample}$$

Total phosphorus content

5 ml of the supernatant clear wet-ashed digested solution was pipette out into a 50 ml volumetric flask. 20 ml of the ascorbic acid and 10 ml of reducing agent (Molybdate reagent: mixture of 2.1 g ammonium molybdate in 200 ml distilled water and 0.2 g antimony sodium tartrate in 200 ml water) was added to the flask. The volume of contents was raised to 50 ml with distilled water and shake well. It was allowed to stand for 1 hour to permit full colour development. The absorbance value of the standard and sample (blue colour) was recorded at 880 nm wavelength using a colorimeter. The corrected absorbance for sample was obtained by subtracting the absorbance of blank. The total phosphorus content was calculated by using the formula:

$$\%P\ of\ the\ Sample = \frac{Corrected\ absorbance \times 0.1}{Weight\ of\ the\ Sample}$$

Total lignin, Cellulose and crude fibre content

Determination of Acid Detergent Fibre (ADF): The weighed air-dried samples were grounded and transferred into extraction tubes. The equal amount of sample was ignited for determining moisture content and ash-free dry mass. 20 ml of acid detergent solution and 0.4 ml deca-hydro-naphthalene was added. The extraction tubes were heated and their content was filtered on pre-weighed tarred Gooch crucible set. Particles were recovered and filtered mat was washed twice with hot water followed by washing with Acetone. Acid detergent fibre was allowed to dry overnight at 105 ^{o}C. Acid detergent fibre (ADF) was calculated using the formula:

$$\% \, ADF = \frac{W_0 - W_t}{W_S} \times 100$$

Where, W_0 = initial weight of oven dried crucible,

W_t = tarred weight of oven dried crucible,

W_S = weight of oven dried sample.

For determination of acid detergent Lignin and Cellulose: The contents of crucibles were covered with cooled 72% H_2SO_4 and stirred with spatula. Crucible was filled hourly with the acid as acid was drained away from crucible. Crucible was kept at 20 – 23 °C. After 3 hours, the content was washed with hot water to make it free from acid. Crucibles were then allowed to dry overnight at 105 °C. The weight of crucible was noted and then crucible was ignited in a muffle furnace at 550 °C and then cooled to 105 °C. Acid detergent Cellulose was calculated using formula:

$$\%Cellulose = \frac{L_A}{W_S} \times 100$$

Where, L_A = loss due to 72% H_2SO_4 treatment

W_S = oven-dried weight of sample

Acid detergent Lignin was calculated using formula:

$$\%Lignin = \frac{L_I}{W_S} \times 100$$

Where, L_I = loss upon ignition after 72% H_2SO_4 treatment

W_S = oven-dried weight of sample

Total Organic Carbon

For estimation of litter TOC, 1 g of oven-dried sample was weighed and transferred into a labelled 100 ml digestion tube. This weight is recorded as W. Then, 2 ml of distilled water was added. It is followed by addition of 10 ml 5% potassium dichromate solution. After this, 5 ml H_2SO_4 was slowly added from a burette and gently swirled the mixture. Then, this mixture was digested at 150 °C for 30 mins. The mixture was then allowed to cool, followed by addition of 50 ml (0.4%) barium chloride and then the mixture was swirled to mix thoroughly. It is left overnight so as to leave a clear supernatant solution. Next day, an aliquot of the supernatant solution was transferred into a colorimeter cuvette, and absorbance of each standard and sample was recorded at 600 nm. The corrected absorbance was calculated by subtracting the mean value of blank samples. The TOC for each sample was calculated by using the following formula:

$$\% \, Organic \; Carbon = \frac{Corrected \; absorbance \times 0.1}{Weight \; of \; initial \; sample \; (g)}$$
